# Liver triacylglycerol content and gestational diabetes: effects of moderate energy restriction

**DOI:** 10.1007/s00125-016-4143-9

**Published:** 2016-11-05

**Authors:** Kenneth Hodson, Chiara Dalla Man, Fiona E Smith, Alison Barnes, Catherine McParlin, Claudio Cobelli, Stephen C Robson, Vera Araújo-Soares, Roy Taylor

**Affiliations:** 1 Institute of Cellular Medicine, Newcastle University, Newcastle upon Tyne, UK; 2 Royal Victoria Infirmary, Newcastle upon Tyne Hospitals NHS Foundation Trust, Newcastle upon Tyne, UK; 3 Newcastle Magnetic Resonance Centre, Newcastle University Campus for Ageing and Vitality, Newcastle upon Tyne, NE4 5PL UK; 4 Department of Information Engineering, University of Padova, Padova, Italy; 5 Institute of Health and Society, Newcastle University, Newcastle upon Tyne, UK

**Keywords:** Gestational diabetes, Insulin resistance, Intrahepatic lipid, Magnetic resonance spectroscopy, Pregnancy

## Abstract

**Aims/hypothesis:**

Women with a history of gestational diabetes mellitus (GDM) have raised liver triacylglycerol. Restriction of energy intake in type 2 diabetes can normalise glucose control and liver triacylglycerol concentration but it is not known whether similar benefits could be achieved in GDM. The aim of this work was to examine liver triacylglycerol accumulation in women with GDM and the effect of modest energy restriction.

**Methods:**

Sixteen women with GDM followed a 4 week diet (5 MJ [1200 kcal]/day). Liver triacylglycerol, before and after diet and postpartum, was measured by magnetic resonance. Insulin secretion and sensitivity were assessed before and after diet. Twenty-six women who underwent standard antenatal care for GDM (matched for age, BMI, parity and ethnicity) were used as a comparator group.

**Results:**

Fourteen women, who completed the study, achieved a weight loss of 1.6 ± 1.7 kg over the 4 week dietary period. Mean weight change was −0.4 kg/week in the study group vs +0.3 kg/week in the comparator group (*p* = 0.002). Liver triacylglycerol level was normal but decreased following diet (3.7% [interquartile range, IQR 1.2–6.1%] vs 1.8% [IQR 0.7–3.1%], *p* = 0.004). There was no change in insulin sensitivity or production. Insulin was required in six comparator women vs none in the study group (eight vs two required metformin). Blood glucose control was similar for both groups. The hypo-energetic diet was well accepted.

**Conclusions/interpretation:**

Liver triacylglycerol in women with GDM was not elevated, unlike observations in non-pregnant women with a history of GDM. A 4 week hypo-energetic diet resulted in weight loss, reduced liver triacylglycerol and minimised pharmacotherapy. The underlying pathophysiology of glucose metabolism appeared unchanged.

**Electronic supplementary material:**

The online version of this article (doi:10.1007/s00125-016-4143-9) contains peer-reviewed but unedited supplementary material, which is available to authorised users.

## Introduction

Gestational diabetes mellitus (GDM) is recognised to be an early manifestation of type 2 diabetes mellitus, with shared pathogenic features [1]. Recently, it has been demonstrated that individuals with type 2 diabetes can durably be returned to non-diabetic glucose control by substantial weight loss depending on initial reduction in liver triacylglycerol content [2, 3]. However, information on liver triacylglycerol content in GDM is limited even in animal models [4].

In type 2 diabetes, both liver triacylglycerol and fasting plasma glucose are normalised within 7 days of a substantial reduction in energy intake [5]. Over a period of weeks, a more moderate reduction of energy intake to 5 MJ (1200 kcal)/day decreases liver triacylglycerol content and plasma glucose [6]. High levels of liver triacylglycerol are known to be present years before the diagnosis of type 2 diabetes [7] and women with previous GDM have markedly elevated liver triacylglycerol levels [8, 9]. As normal pregnancy is associated with a greater than twofold increase in plasma triacylglycerol levels [10], a physiological rise in liver triacylglycerol would be expected during pregnancy as these variables are usually closely associated [11]. This may be exaggerated in pregnancies complicated by GDM.

Food restriction in pregnancy, as a way to improve adverse metabolic factors, understandably raises concerns. Many of these concerns are unfounded [12] and, conversely, both obesity and gestational diabetes are known to confer substantial risks [13, 14]. Meta-analysis has shown that weight loss in pregnancy in healthy women reduces the incidence of GDM and also pre-eclampsia, gestational hypertension and preterm birth, with no effect on fetal growth [15]. However, current guidelines do not recommend weight loss during pregnancy [16]. Further, there is little information on how accepting women with GDM would be towards receiving specific advice to decrease energy intake.

The primary aims of this study were to define the extent of liver triacylglycerol accumulation during pregnancy in women diagnosed with GDM and the effect upon this of modest energy restriction. The metabolic effects and acceptability of energy restriction in GDM were also examined.

## Methods

### Study population

Between January 2015 and August 2015, 16 women between 21 weeks and 34 weeks gestation (mean 27 ± 3.3 weeks) with a singleton pregnancy were recruited from the antenatal clinic at the Royal Victoria Infirmary, Newcastle upon Tyne following a positive 75 g OGTT (fasting glucose ≥5.5 mmol/l, 2 h glucose ≥7.8 mmol/l) [17]. In accordance with National Institute for Health and Care Excellence (NICE) guidelines the presence of one or more of the following necessitated an OGTT [18]: BMI above 30 kg/m^2^, previous baby weighing more than 4.5 kg, previous GDM, family history of diabetes and ethnic minority with a high prevalence of diabetes. Women with multiple pregnancy or contraindication to magnetic resonance scanning (ferromagnetic implant, claustrophobia, abdominal circumference >102 cm) were excluded. To compare weight change and pregnancy outcomes, each participant was matched with the two best comparators with GDM available from the Caldicott approved hospital maternity database according to age (within ±5 years, mean difference 0.5 ± 3.3 years), BMI (within ±6 kg/m^2^, mean difference 1.2 ± 2.9 kg/m^2^), parity (nulliparous or multiparous) and ethnic origin. Data regarding weight change, fetal growth, home blood glucose monitoring and treatment were obtained for the matched participants over a similar 4 week gestational window. The study participants and the comparators were both identified by initial screening followed by a positive OGTT using the above criteria. The comparator group were reviewed once by a dietitian, given a dietary information leaflet and were taught how to carry out home blood glucose monitoring; glycaemic control was overseen by a diabetes specialist midwife.

The Wellbabe (Weight Loss Looking for Baby and Mother’s Better Outcomes) study was approved by the Newcastle and North Tyneside Ethics Committee (14/NE/1085) and all women gave written informed consent. The study was registered with the ISRCTN (http://isrctn.org 17505466).

### Protocol

Women were invited to participate at the first clinic visit after diagnosis of GDM. A magnetic resonance spectroscopy (MRS) scan and standardised meal test were performed before and after the 4 week hypo-energetic diet. Fetal growth scans, measuring abdominal circumference [19], were conducted at 28, 32 and 36 weeks’ gestation and data regarding weight and home blood glucose monitoring were collected. In the light of data obtained, further ethical permission was obtained to carry out postpartum liver triacylglycerol measurements and fasting blood tests between 12 weeks and 28 weeks after delivery.

### Dietary intervention

The 5 MJ (1200 kcal)/day diet (50% carbohydrate, 25% protein, 25% fat) was designed to limit energy intake while ensuring nutritional adequacy (food portion plan plus calcium-containing pregnancy multivitamins) during pregnancy. Specific dietary advice was provided during a face-to-face consultation, delivered by trained team members, following the standardised meal test at the first visit to the clinic. The rationale for the diet was explained and motivation, facilitators and barriers to implementation of the dietary changes were explored. The diet portion plan was explained in full and a supporting booklet was provided (see electronic supplementary material [ESM]) along with a sample 7 day meal plan and suggested recipes. Each participant’s usual energy intake was reviewed and modified to match the 5 MJ (1200 kcal) portion plan. Where necessary the portion plan was changed to reflect an individual’s food preferences. A portion cup was provided to measure appropriate amounts of breakfast cereals, rice and pasta. MyFitnessPal (MFP) (Under Armour, Baltimore, MD, USA), a smartphone application, was used to record dietary intake. Women consented to sharing their dietary and glycaemic control data with the research team (KH and AB) so that progress could be monitored daily and support and advice given via MFP messaging or telephone call accordingly (based on MFP data assessment by the study team). Targets for home blood glucose monitoring were fasting glucose <5.5 mmol/l and 1 h postprandial glucose <7.8 mmol/l. Metformin and/or insulin were started if glucose levels were persistently above target. Following completion of the 4 week dietary plan, KH provided a revised portion plan for the remainder of the pregnancy. In most cases the women were advised to avoid weight gain and to continue on ∼1500 kcal/day, although this was individualised according to weight loss and glycaemic control.

### Magnetic resonance protocol

Studies were performed using a Philips 3 Tesla Achieva whole-body scanner (Philips Medical Systems, Best, the Netherlands) using a Philips multi-channel flex coil for ^1^H imaging and spectroscopy. To avoid pressure on the inferior vena cava, participants were positioned with a left pelvic tilt. Scout images of the maternal abdomen were acquired to guide identification of the volume of interest within the liver. ^1^H spectroscopy comprised acquisition of point resolved spectroscopy-localised spectra at six echo times (repetition time = 2.8 s; echo time = 36, 50, 75, 100, 125 and 150 ms; spectral width = 2 kHz; 2000 data points) from a 3 cm × 3 cm × 3 cm voxel positioned in the liver to avoid large vessels.

Spectra were processed using the Java-based magnetic resonance user interface (jMRUI version 3.0) [20, 21] and peak areas were determined using the AMARES non-linear least square fitting algorithm [22]. Resonances of water at 4.7 ppm and the CH_2_ methylene peak at 1.3 ppm in ^1^H spectra were quantified. The mean spin–spin relaxation time (T2) was determined for each peak by fitting a mono-exponential to the data. Signal amplitude at an effective echo time of zero was determined, and the amplitude used to obtain the liver triacylglycerol fraction value. The percentage of triacylglycerol was calculated from the ratio of signal amplitude triacylglycerol divided by total signal amplitude from water plus triacylglycerol × 100. The upper limit of normal for a US population of mixed-sex, multi-ethnic participants aged 30–65 years has been defined as 5.5% [23].

Liver triacylglycerol content was assessed at baseline and after the 4 week hypo-energetic diet. Participants continued on the diet until the second visit to the clinic.

### Standardised meal test

Prior to metabolic testing, women were asked to avoid vigorous exercise and to fast overnight. Participants were transported to and from the Newcastle Magnetic Resonance Centre by taxi. A standardised breakfast was eaten over 10–15 min (two Weetabix [wholewheat cereal biscuits], 200 ml semi-skimmed milk, 200 ml orange juice, a white bread roll, 20 g strawberry jam and 10 g margarine, totalling 2.4 MJ [575 kcal; 72% carbohydrate, 15% protein, 13% fat]). Blood samples for measurement of glucose, insulin and C-peptide were taken via an intravenous cannula at 0, 10, 20, 30, 60, 90,120 and 180 min. HbA_1c_, lipids (triacylglycerol, total cholesterol, HDL-cholesterol and LDL-cholesterol), fasting NEFA and urea and electrolytes were measured and liver function tests and full blood count were performed [24, 25]. The participants were sitting comfortably during the meal test, with intermittent brief walking as desired. The meal test was carried out at baseline and at the end of the hypo-energetic diet.

Insulin sensitivity in the fasted state was calculated from fasting glucose and insulin concentrations using the HOMA2 index (available from www.dtu.ox.ac.uk/homacalculator/, accessed 21 June 2016 [26].) The oral glucose minimal model was used to analyse insulin sensitivity (S_I_) during the meal [24, 25]. Beta cell insulin secretion was calculated from the oral C-peptide minimal model [24, 25].

### Home blood glucose monitoring

Women were supplied with a Bayer Contour meter (Basel, Switzerland). They were asked to measure glucose levels daily before breakfast (fasting) and 1 h after their main meal. Data were relayed to KH daily either through MFP or by telephone. The need for metformin and/or insulin was assessed as part of routine management.

### Qualitative study

A semi-structured interview was conducted with each participant by an independent research midwife (CM) who was experienced in qualitative methodology. An interview schedule was developed using the Theory Domain Framework [27] to explore motivation to engage in the diet, beliefs about consequences and emotions (e.g. fears) among other domains. The full qualitative study analysis will be published separately.

### Statistics

Data were analysed in SPSS V21.0 (IBM, Armonk, NY, USA). Continuous variables are expressed as mean ± SD. Continuous data were compared using the two-tailed paired Student’s *t* test and ANOVA when there were multiple groups. Non-parametric continuous data were compared using Wilcoxon’s signed-rank test. Significance was set at *p* < 0.05.

A power calculation was undertaken based upon change in liver triacylglycerol. This was based on a previous study from our institution in participants with type 2 diabetes as there were no published data on liver triacylglycerol in human pregnancy [5]. In our previous study liver triacylglycerol fell from 12.8 ± 2.4% to 4.8 ± 4.2% over a 4 week period. Given that the degree of energy restriction in the present study was 60% less than in the previous study, and that fall in liver triacylglycerol is proportionate to reduction in energy intake, we assumed that the reduction in liver triacylglycerol would be 60% of that previously reported. Thirteen participants would be required to demonstrate an absolute fall in liver triacylglycerol of 4.8% (assuming SD of 4.2 and baseline level of 12%) with 95% power at the 5% significance level.

## Results

### Patient demographics

Sixteen women were recruited. Two women dropped out (during weeks 1 and 2) citing pressure of time and social circumstances. One participant was unable to undergo magnetic resonance (claustrophobia) but underwent all other aspects of the protocol. To compare the clinical effect of the hypo-energetic diet with that of standard management, matched comparators with GDM were identified from the maternity database (*n* = 28; complete data available on 26) (Table 1).

**Table 1:** Baseline characteristics of participant and comparator groups

Characteristic	Participants (*N* = 14)	Controls (*N* = 26)
Age (years)	31.5 ± 4.6	30.9 ± 4.4
Weight (kg)	93.1 ± 13.9	90.5 ± 17.1
Height (cm)	164 ± 6.3	163 ± 5.8
BMI (kg/m^2^)	34.6 ± 4.1	34.1 ± 6.1
Nulliparous	8 (57)	15 (58)
History of GDM/type 2 diabetes	12 (86)	N/A
OGTT (mmol/l)		
0 min	5.0 ± 0.8	5.1 ± 1.0
120 min	8.5 ± 0.6	8.9 ± 1.8

Data are shown as means ± SD or *n* (%)

All participants and controls were of white British ethnicity

### Weight change

During the hypo-energetic diet, participants lost a mean of 0.4 ± 0.4 kg per week during the intervention compared with a weight gain of 0.3 ± 0.3 kg per week in the comparator group (*p* < 0.001). The total weight loss in participants was 1.6 ± 1.7 kg compared with a total gain of 1.4 ± 1.2 kg in comparators. Six participants lost more than 2 kg (2.1–5.6 kg), five lost 0.3–1.2 kg and three participants put on weight (0.2–0.5 kg).

### Liver triacylglycerol

**Fig. 1:**
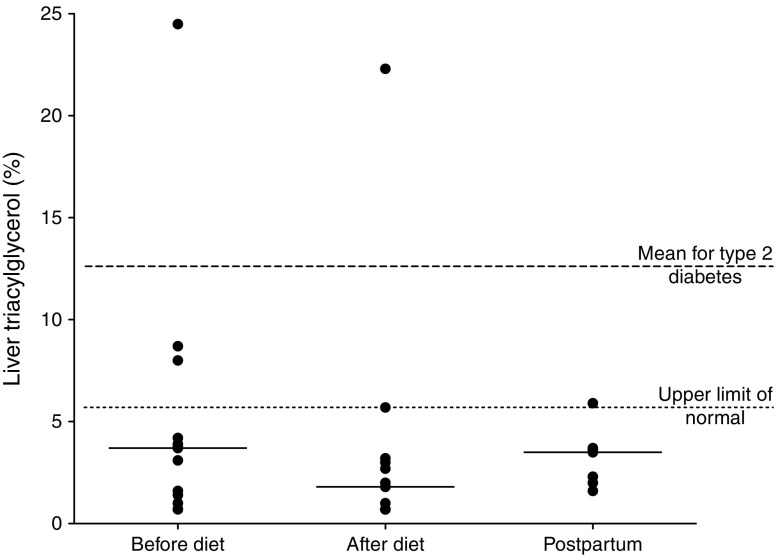
Percentage liver triacylglycerol before and after dietary intervention (*n* = 13) and postpartum (*n* = 7). Circles show individual values; bars show the median. *p* = 0.004 before vs after intervention

Before dietary intervention, at gestational age 21–34 weeks, median liver triacylglycerol was 3.7% (interquartile range [IQR] 1.2–6.1%). After 4 weeks of dietary intervention the median liver triacylglycerol was approximately halved to 1.8% (IQR 0.7–3.1%; *p* = 0.004). Individual data are shown in Fig. 1. One woman had very high pre-diet liver triacylglycerol levels (>20%); repeat analysis excluding this individual did not change the statistical significance (3.4% [IQR 1.1–4.1%] pre-diet, 1.4% [IQR 0.7–2.9%] post-diet; *p* = 0.006).

Measurement of liver triacylglycerol was repeated at 12–28 weeks postpartum; median levels were similar to those seen pre-diet (pre-diet 3.7% [IQR 1.2–6.1%], postpartum 3.5% [IQR 2.0–3.7%], *p* = 0.81).

### Standardised meal test

Fasting plasma glucose remained unchanged after the dietary period (4.3 ± 0.5 mmol/l vs 4.4 ± 0.7 mmol/l, *p* = 0.49). The HOMA2 index was similar before and after dietary intervention (1.3 ± 0.4 vs 1.4 ± 0.4, *p* = 0.47) and was lower postnatally (0.6 ± 0.2, *p* < 0.01). The postprandial glucose concentration curve was similar before and after dietary intervention. Glucose peaked at 60 min at a concentration of 8.2 ± 1.0 mmol/l before the diet and 8.4 ± 1.5 mmol/l after the diet. There was no statistically significant difference between fasting insulin or C-peptide before and after the diet (38 ± 19 vs 48 ± 23 pmol/l insulin, respectively, *p* = 0.12; 0.61 ± 0.19 vs 0.65 ± 0.10 nmol/l C-peptide, respectively, *p* = 0.36). Acute insulin secretion did not change during the standard meal test after the diet (Φ_total_ 62 ± 18 vs 58 ± 12 [×10^−9^]/min, *p* = 0.52). Insulin concentrations peaked at 60 min (493 ± 148 pmol/l before diet vs 513 ± 239 pmol/l after diet, *p* = 0.69). C-peptide levels peaked at 90 min before diet (3.0 ± 0.9 nmol/l) and at 120 min after the diet (3.7 ± 0.9 nmol/l). There was no change in post-meal S_I_ before and after diet (14.3 ± 6.0 vs 13.4 ± 7.6 × 10^−5^ dl kg^−1^ min^−1^ [pmol/l]^−1^, *p* = 0.55).

### Lipid profile

**Fig. 2:**
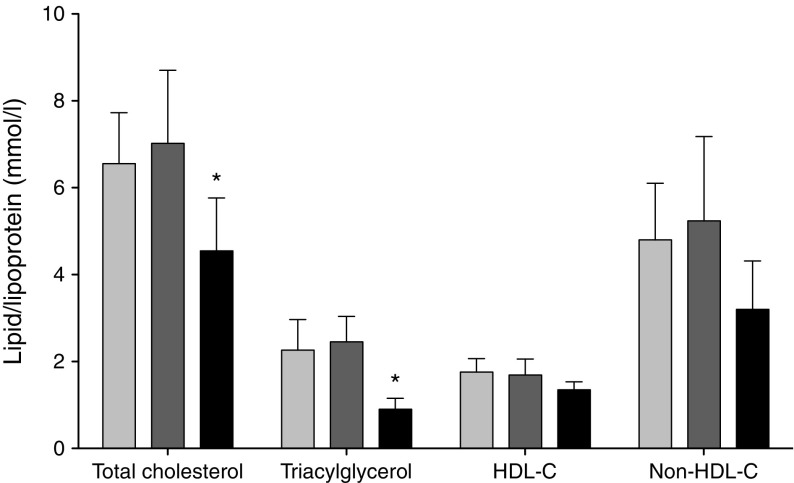
Plasma cholesterol and triacylglycerol lipoproteins in women with GDM before (light grey bars) and after (dark grey bars) dietary intervention (*n* = 14) and postpartum (black bars) (*n* = 7). Data are means ± SD. *p < 0.05 before diet vs postpartum

Lipid profiles before and after diet and during the postnatal period are shown in Fig. 2. The pregnancy-associated increase in plasma triacylglycerol was unchanged by the hypo-energetic diet. Similarly, there was no difference in HDL-cholesterol and non-HDL-cholesterol levels before and after the diet. Mean triacylglycerol and total cholesterol levels fell after delivery (*p* < 0.05 for both).

### NEFA

There was no significant difference in fasting NEFA before and after intervention (0.42 ± 0.21 vs 0.48 ± 0.25 mmol/l; *p* = 0.18).

### Liver function tests

Median alanine aminotransferase (ALT) was 18 U/l (IQR 14–25 U/l) before dietary intervention and 24 U/l (IQR 20–32 U/l) after. Two participants had an elevated ALT (62 and 93 U/l) pre-diet and one had an ALT that rose from 26 U/l before intervention to 83 U/l following the diet. The women with elevated ALT had negative investigations for viral hepatitis. In these two participants ALT was monitored throughout pregnancy and returned to normal limits after delivery.

### Glucose control

None of the hypo-energetic diet group required insulin therapy compared with six of the 26 women in the comparator arm. Two participants in the diet group required metformin therapy (weight loss 0.3 kg and 1.2 kg) compared with eight of the comparators, six women requiring both metformin and insulin in the comparator population.

Despite the pharmacotherapy, home monitoring during the 4 week intervention period showed similar mean blood glucose levels for the diet and comparator groups respectively (fasting 4.9 ± 0.6 vs 4.9 ± 1.0 mmol/l; postprandial 6.6 ± 0.8 vs 6.6 ± 0.9 mmol/l). Mean HbA_1c_ did not differ between groups (5.2 ± 0.6% [34 ± 4 mmol/mol] vs 5.3 ± 0.8% [34 ± 5 mmol/mol] for the diet and comparator groups respectively, *p* = 0.89).

### Maternal and fetal outcomes

All women in the dietary intervention group expressed positive thoughts about the experience of decreasing energy intake during pregnancy. There was no difference in mode of delivery between participants and comparators. None of the women had shoulder dystocia or a third-degree tear. No difference in the rate of increase in fetal abdominal circumference was observed when comparing the diet and comparator groups (ESM Fig. 1). There was no difference in birthweight between participant and comparator groups (Table 2). One baby in the study group was admitted to the special-care baby unit for chylothorax, detected at 36 weeks’ gestation. Excluding this baby, APGAR scores ranged from 7 to 9 at 1 min and from 9 to 10 at 5 min. No babies had neonatal hypoglycaemia.

**Table 2:** Maternal and fetal outcomes

Outcome	Participants (*N* = 14)	Controls (*N* = 26)	*p* value
Mean gestation at delivery (weeks)	38	38	
Spontaneous vaginal delivery	6 (54%)	10 (38%)	
Instrumental delivery	1 (10%)	5 (19%)	
Elective Caesarean section	2 (18%)	6 (23%)	
Emergency Caesarean section	2 (18%)	5 (19%)	
Birthweight (g)	3360 ± 277	3361 ± 398	0.99
Average birthweight (centile)	54 ± 26	52 ± 25	0.84
SCBU admission	1	2	

Data are shown as means ± SD, *n* or *n* (%)

SCBU, special-care baby unit

## Discussion

The present study suggests that GDM is not typically characterised by high levels of liver triacylglycerol. The hypo-energetic diet brought about a weight loss of 0.4 kg/week and halved liver triacylglycerol content. Over the 4 week treatment period there was no change in insulin secretion in response to a test meal. The diet was well tolerated, resulting in glycaemic control equivalent to that achieved using conventional management (insulin and metformin as required).

The observation of normal liver triacylglycerol levels did not match the expectation arising from analysis of the literature. Previous studies have demonstrated that non-pregnant women with a history of GDM have elevated liver triacylglycerol levels [8, 9] and have a greater risk of non-alcoholic fatty liver disease in later life [28]. Given that excess intrahepatic triacylglycerol is an important underlying factor in the development of type 2 diabetes, with average levels of 12.8 ± 2.4% [3, 7], it was anticipated that increased fat would be observed in women with newly diagnosed GDM. Further, raised liver triacylglycerol levels in type 2 diabetes are associated with raised plasma triacylglycerol [29], and plasma triacylglycerol levels are increased to a greater extent in GDM than in non-diabetic pregnancy [29]. Abnormal lipid metabolism appears to have a central role in GDM [10]. The present data are the first in vivo magnetic resonance liver triacylglycerol measurements to be reported during human pregnancy. The observation of apparently normal liver triacylglycerol content in most participants raises the possibility that liver triacylglycerol content may decrease during pregnancy despite increases in plasma triacylglycerol, and that levels which are normal in the non-pregnant state may be associated with GDM. It is interesting to note that liver triacylglycerol, but not plasma triacylglycerol, decreased following hypo-energetic dieting. This is likely to reflect the physiological adaptation of pregnancy, with requirement for a sharp increase in plasma triacylglycerol after the first trimester, and change in nutritional state is unlikely to change this.

The present data raise the possibility that liver fat levels fall reciprocally with elevation of plasma triacylglycerol. This hypothesis can be tested in a future study. It is consistent with ultrasound findings of an association between the presence of liver steatosis in early pregnancy and the subsequent risk of GDM [30] and also the association of raised ALT with risk of GDM [31]. However, in the aforementioned study, liver ultrasound was performed between 11 and 14 weeks, before the onset of either raised plasma triacylglycerol or significant insulin resistance. In the present study we did not observe any marked change in liver triacylglycerol after pregnancy, although it is not known how long any pregnancy-associated change in liver triacylglycerol may take to return to non-pregnancy levels. Now that the safety of MRS in pregnancy is accepted, further work is required to establish the physiology of liver triacylglycerol in non-diabetic pregnancy and to compare differences in GDM. To our knowledge, there are no quantitative studies of human or animal liver fat during normal pregnancy. It is established that a very-high-fructose diet during pregnancy is associated with histologically assessed steatosis in animal models [4].

The study group were representative of the wider population of women with GDM. Age and BMI were similar to those of participants in larger studies of GDM [14, 32]. HbA_1c_ in the study group (5.3 ± 0.4% [34 ± 4 mmol/mol]) was similar to that at the time of diagnosis in all women recorded in the Newcastle GDM database (5.5 ± 0.8% [36 ± 9 mmol/mol]). The group was predominantly white British, reflecting the population of the North East of England.

The time course of return of normal first-phase insulin secretion for people with type 2 diabetes during a very-low-energy diet has been defined [5]. Even at 2.5–3.3 MJ (600–800 kcal)/day, 8 weeks was required for normal insulin secretion to be restored and at 4 weeks improvement was modest. The present study necessarily used a less severely restricted diet of 5 MJ (1200 kcal)/day and, as a first step, this was advised for 4 weeks only. The lack of change in insulin secretion following the test meal is therefore not unexpected. Further work is required to establish whether the insulin secretory abnormality in GDM [33], being of short duration, is more readily reversed than that of type 2 diabetes.

Dietary weight loss during pregnancy is viewed with caution by many obstetricians, even though obesity is a major risk factor for macrosomia and associated adverse outcomes. The benefits of minimising weight gain during pregnancy, in the present era of steady weight gain during adult life, were first reported several years ago [34]. This is especially relevant in GDM [14, 32, 35]. A clear decrease in energy intake has been achieved on a whole-clinic basis by Asbjornsdottir and colleagues who achieved a decrease in median weight gain during pregnancy from 12.1 to 3.7 kg [36]. This was associated with decreases in large-for-gestational-age infants (39% to 12%) and perinatal morbidity (71% to 35%). At the time when GDM is diagnosed there is likely to be increased motivation to decrease energy intake. All 14 women who completed the study reported that they were comfortable with the explanation of likely benefit for their baby. The present study is unique in demonstrating the effectiveness and acceptability of modest weight loss at the time of diagnosis of GDM. A randomised study of dietary weight loss is now required.

Several practical features of the dietary intervention merit discussion. The reasons why weight loss in pregnancy was believed to be important for fetal health were carefully explained to each woman. Diets were individualised according to preferred eating habits and women were asked to discuss this with family and friends. The use of current smartphone technology encouraged engagement with the diet and regular communication with the study team. Women reported that this immediacy of communication facilitated dietary compliance and allowed access to medical advice for management of their blood glucose levels. The dietary intervention was more intensive than conventional intermittent clinic review, although halving of gestational weight gain has previously been reported using monthly dietetic consultations [37].

The number of women studied was small, but the participants were representative of the wider population with GDM. The study was large enough to demonstrate a highly statistically significant difference in weight loss between intervention and comparator groups. However, due to the small sample size it was not possible to adjust for other factors that may differ between the groups, nor was it possible to subanalyse the group (for example, to study the outcome of those who lost more weight than others). As women were advised of the diagnosis of GDM and of the aims of the study at a clinic visit several days before the baseline measurements, the baseline necessarily reflects an initial dietary intervention (mean fasting glucose falling from 5.0 mmol/l to 4.3 mmol/l). Avoidance of insulin therapy is associated with major benefit in simplifying peripartum obstetric management as well as minimising weight gain, personal inconvenience and use of healthcare resources. Even so, most women were in the lower range of plasma glucose concentration for diagnosis of GDM and it will be important to study women with higher presenting blood glucose levels. Size restrictions within the magnetic resonance scanner preclude women with an abdominal circumference greater than 102 cm from taking part in such studies. However, no woman in this study was excluded on this criterion. Women with the highest BMI might be expected to have the strongest association between GDM and hepatic steatosis and to experience a more dramatic effect of energy restriction on the liver. However, recent data on the lack of association between raised ALT and risk of GDM in more obese women suggest that this is unlikely [31].

The present study highlights the important question of liver triacylglycerol physiology in normal and GDM pregnancy and provides data for informing the design of further studies. Additionally, there is a need for a prospective randomised therapeutic study of dietary weight loss from the time of diagnosis of GDM.

## Electronic supplementary material

Below is the link to the electronic supplementary material.

ESM (PDF 562 kb)

## Abbreviations

ALT: Alanine aminotransferase
GDM: Gestational diabetes mellitus
IQR: Interquartile range
MFP: MyFitness Pal
MRS: Magnetic resonance spectroscopy
