# Supplementary material for: Liver triacylglycerol content and gestational diabetes: effects of moderate energy restriction
Source: Diabetologia. 2016 Nov 5;60(2):306–13. doi: 10.1007/s00125-016-4143-9 (PMC6518369; doi:10.1007/s00125-016-4143-9)
Supplement: Supplementary file 1 — (PDF 562 kb) [file 125_2016_4143_MOESM1_ESM.pdf]

WELLBABE Study

1200 Kcal Diet Plan

Gestational Diabetes and Food

Your doctor or diabetes midwife will have explained the importance of regulating your blood glucose levels when you have gestational diabetes. This can often be achieved by making dietary changes, with additional medications if required.

Most foods are a combination of protein, fat and carbohydrate in different proportions. It is the carbohydrate in foods which has the most effect on blood glucose levels. This is because carbohydrate is broken down into glucose during digestion. The glucose then enters the blood.

Carbohydrates include starchy foods such as bread, rice, potatoes, pasta and cereals (including flours), added sugars (e.g. sucrose), and also natural sugars found in fruit and milk. They do not need to be avoided completely; however it is important to eliminate foods and drinks which contain lots of added sugar:

1. Cut out sugary pop, squashes and juices. Swap to diet/zero/slimline pop and no added sugar squashes, as these do not raise blood glucose levels. Most flavoured waters are also a good choice, but check the ingredients list and labels, as a few do contain added sugar.
2. Limit fruit juice to 1 small glass (150ml) per day and have this with food. Avoid smoothies and milkshakes which contain lots of sugar.
3. Use a low calorie sweetener in place of added sugar in hot drinks and on cereals e.g. Canderel, Splenda, Hermesetas, Stevia.
4. Jelly contains lots of added sugar (31 sugar cubes in a block of jelly). Swap to sugar free jelly which contains no sugar at all. No Added Sugar Angel Delight is also available.
5. Choose tinned fruit in natural juice rather than in syrup or light syrup.
6. Buy reduced sugar jam and marmalade.
7. Diet/light fruit yogurts use sweetener instead of sugar.
8. Avoid sweets (jellies, boiled sweets, toffees etc.) and ice lollies. Sugar free mints and chewing gum are fine to have (in moderate amounts as the sweeteners used can have a laxative effect)

How else can I keep my blood glucose levels in target?

- Spread your food (especially your carbohydrates) across the day, eating little and often

- Try to be as active as you can within your limitations. A 20 minute walk after a meal can help to reduce blood glucose levels more quickly.

- Manage your weight during and after pregnancy – this is the aim of the WELLBABE study you are participating in.

How will weight loss help me and my baby?

- Weight loss helps insulin to work more efficiently to remove glucose from your blood. By improving your blood glucose levels your baby is less likely to grow bigger than expected, making delivery easier. It is also less likely that your baby will have a low blood sugar (hypo) after delivery if your blood glucose is well controlled.

- At a lower weight you are less likely to develop Type 2 Diabetes in the future, or develop gestational diabetes again in future pregnancies.

- Continuing the healthy changes you have made after pregnancy can help the whole family to eat a healthy diet and maintain a healthy weight – this is important as Type 2 Diabetes does run in families.

Before you Begin

It is helpful to think about what you hope to gain (what it would mean to you) from following the diet plan/losing weight, and also what challenges might arise during the 4 weeks of the diet.

Benefits	Difficulties
E.g. able to fit into clothes better, will be less breathless when climbing the stairs	E.g. I tend to eat when I'm stressed/bored/upset, there are always cakes/biscuits around at work, I have a birthday in 2 weeks time

For each difficulty you have identified, think about how you could manage this in a different way i.e. without resulting in eating more than planned.

Difficulty	Plan
E.g. I eat when I am stressed	Manage the stress by talking to a friend or letting it out in another way (punchbag/a good shout in the garden)

What will I eat on the 1200 kcal diet?

The diet plan consists of set quantities of foods from 6 different food groups. This will provide you with a varied and balanced low calorie diet which is designed to provide maximum nutrition for you and your baby. You will also be taking a pregnancy multivitamin while following the diet to supplement the nutrition from foods.

Food Group	Number of Portions Daily
Starchy Foods	4
Protein	2
Milk and Dairy	2
Fruit	1
Vegetables	4
Fats & Oils	2

You can distribute these foods as you wish across the day to suit your preferred eating pattern. The lists on the following pages give portion sizes for the different food groups.

Other Factors

- Drink 2 litres of sugar/calorie free fluids per day while following the diet plan. This includes water, diet fizzy pop, no added sugar squash, decaffeinated tea and coffee, herbal teas. Limit caffeinated tea/coffee to 2 mugs (4 cups) per day.
- Avoid calorie containing drinks (sugary pop and squash, smoothies, milkshakes, lattes etc.). Alcohol should be avoided as per standard advice for pregnancy.
- You will need to avoid many higher calorie snacks to remain within the 1200 calorie limit. This includes sweets, chocolate, biscuits, crisps and cakes. There are snack ideas on page 9 of this booklet.

Portion Sizes

Starchy Foods (Allowance 4 portions per day)

100kcal per portion

**1 Portion:**	
1 medium slice of bread or toast	30g uncooked rice, pasta or bulgur wheat
1 sandwich thin	75g cooked rice or pasta
1 mini pitta bread	4 new potatoes in skins (150g)
1 small chapatti/1 flour tortilla	2 egg-sized old potatoes (boiled or mashed)
1 ½ Weetabix	100g cooked sweet potato
1 shredded wheat	8 oven chips
1 crumpet or scotch pancake	2 crispbreads
30g porridge oats (uncooked)	3 plain crackers
25g breakfast cereal (3 tblspns)	2 small Yorkshire puddings

**2 Portions:**
45g muesli (not granola)
1 full size pitta bread
1 bagel or English muffin
1 medium bread bun (50g)
1 nest of noodles
1 medium jacket potato (200g cooked weight)
1 individual packet (110g) couscous

**Choose these starchy foods to help keep you fuller for longer:**
Wholegrain breakfast cereals, porridge oats (not instant), wholegrain/granary bread, sourdough bread, sweet potato, new potatoes in skins, basmati rice, pasta, noodles.

Note: Pulses also contain slow release carbohydrate and are a good choice to fill you up without adding lots of calories.

Protein Foods – Meat, Fish, Eggs, Beans & other non-dairy sources of protein (Allowance 2 portions per day)
150kcal per portion

**1 Portion:**	
2 medium eggs	3 rashers grilled back bacon
1 small grilled chicken breast (100g)	2 small slices corned beef (70g)
1 fillet of baked/steamed cod (150g)	4 sandwich-sized slices roast ham (120g)
1 small grilled lamb chop or lamb steak (60g)	150g Quorn / Soya / Tofu
2 thin slices roast meat (75g)	150g cooked prawns
½ tin baked beans in tomato sauce (150g)	100g smoked salmon
1 tin tuna in brine or spring water	45g (1 fillet) smoked mackerel
½ tin salmon in brine	200g cooked/tinned kidney beans, chickpeas, lentils etc.
1 small grilled tuna/salmon steak (100g)	1 tin sardines in brine or tomato sauce
1/3 small pack lean beef mince (75g)	3 grilled fish fingers

Avoid processed meat products (burgers, sausages, pies, chicken kiev etc.) while following the diet plan.

Milk & Dairy Foods (Allowance 3 portions per day)

100kcal per portion

1 Portion:	
200ml semi skimmed milk allowance for cereal and tea/coffee (or unsweetened soya milk)	300ml skimmed milk or unsweetened rice milk
150g (small pot) diet/light yogurt or fromage frais	50g low fat cheese spread (2 matchboxes)
Small matchbox size piece of cheese (25g full fat, 35g reduced fat)	75g cottage cheese/100g reduced fat cottage cheese*
125g natural yogurt/ 160g low fat natural yogurt/190g fat free natural yogurt	

*Should be made with pasteurise milk

Vegetables (Allowance 4 portions per day)

30kcal per portion

1 PORTION:	
3 heaped tablespoons (80g) raw or cooked vegetables (fresh, frozen or tinned)	Use a wide variety of vegetables, choosing different colours to give a range of nutrients.<br><br>Avoid avocados while following the diet plan.<br><br>Note: potatoes and sweet
2 heaped tablespoons peas, sweetcorn or broad beans	
1 dessert bowl fresh salad	
200ml tomato juice	

100g (1/4 tin) tinned tomatoes	potatoes count as starchy foods rather than vegetables
80g tomato salsa	
8 olives	

Fruit (Allowance 1 portion per day)

60kcal per portion

1 PORTION:	
1 medium piece of fruit (e.g. apple, pear, orange etc.)	1 slice of large fruit (melon, pineapple)
1 small banana	Small handful grapes, cherries or berries
2 small fruits (e.g. plums, satsumas etc.)	3 tablespoons fruit salad (fresh or tinned in juice)
4 dried prunes or apricots	1 ½ tablespoons raisins

150ml pure unsweetened fruit juice can count as a maximum of one portion of fruit per day, however fresh/tinned/frozen whole fruits are preferable as they are higher in fibre.

Fats, Oils & Spreads (allowance 2 portions per day)

60kcal per portion

1 PORTION:	
2 tspn (10g) marg/spread	1 ½ tspn salad dressing (fat free dressing is not counted)
4 tspn (20g) light marg/spread	3 tspn soured cream (30g) or 6 tspn if reduced fat
2 tspn vegetable/olive oil (low calorie spray oil not counted)	2 tspn crème fraiche (20g) or 4 tspn if reduced fat
4 tspn salad cream or light mayo (20g)	3 tspn double cream (12g) or 8 tspn single cream (30g)
2 tspn mayonnaise (10g)	175ml gravy (made with granules, no fat added)

10g nuts e.g. 10 peanuts/almonds, 6 cashews (avoid honey roasted)	40g (4tspn) houmous or 8 tspn reduced fat houmous
10g (1 tspn) low fat peanut butter	45ml ketchup or brown sauce
1 1/2 tspn jam or marmalade (20g)	4 tspn reduced sugar jam or marmalade

Snacks

The following are snack ideas for between meals. Remember that snacks still count towards your overall calorie allowance so should be part of your meal plan, not added as extras.

- Vegetable sticks and tomato salsa dip (1 veg portion)
- 1 crumpet with 2 tspn spread (1 starchy, 1 fat portion)
- 2 crispbreads or oatcakes with 50g low fat cottage cheese (1 starchy, ½ dairy portion)
- 1 piece of fruit (1 fruit portion)
- 1 small slice of malt loaf (35g) (1 starchy portion)
- Diet/light yogurt (125 - 150g pot) (1 dairy portion)
- 1 matchbox-sized piece of cheese (chopped into cubes) and 8-10 pickles e.g. pickled onions, gherkins (1 dairy portion, 1 veg portion)
- 1 mini pitta bread or 5 breadsticks with houmous (1 starchy, 1 fat portion)
- 1 cup (25g) plain or salted Popcorn (1 starchy portion)
- 1 low calorie packet of crisps (<100kcal/bag) e.g. Quavers, Skips, French Fries etc. (1 starchy portion)
- 2 biscuits (<50kcal each) e.g. ginger biscuit, nice, malted milk, rich tea, shortcake, iced ring, small cookie, pink wafer, jaffa cake (1 starchy portion)
- Sugar free jelly (not counted)

- Low calorie hot chocolate e.g. options/highlights made with milk (reduce milk portion by 50ml) 1 dairy portion)
- 10g nuts (e.g. 10 peanuts) + 1 raisin snack box (1 fat, 1 fruit)
- 8 olives (1 protein, 1 veg)

Family Meals and Recipes

You may be cooking for others in the family, so it's helpful to be able to work out the number of portions from each food group in the whole recipe using the portion lists on pages 3-6. You can then work out how many portions are in one serving.

Here is an example:

Chilli con Carne (serves 4)

Recipe Ingredient	Food Group	Portions in recipe	Portions per person
Vegetables (1 large onion, 1 red pepper)	Vegetables	2	0.5
450g lean minced beef	Protein	6	1.5
400g tin chopped tomatoes	Vegetables	2	0.5
400g tin red kidney beans	Protein	2	0.5
600g cooked rice	Starchy foods	8	2
Not counted: 2 garlic cloves, 1 tspn chilli powder, tomato puree, black pepper			

Per serving, this meal provides 1 portion of vegetables, 2 portions of protein and 2 portions from the starchy foods group.

Shop-Bought Meals (Ready Meals, Sandwiches etc.)

1. Note that processed foods tend to be less filling, so try to cook your own meals wherever possible, or add extra vegetables to fill you up.
2. As a guide, choose products which contain under 400kcal per portion.
3. Look out for healthy option ranges as these tend to be lower in calories, fat and salt.
4. Compare front of pack Traffic Light labelling, and choose options with fewer reds and more greens, especially for saturated fat. This type of fat increases insulin resistance, making it more difficult for your insulin to control your blood glucose levels.

As an example:

Meal 1 (Shepherd's Pie)

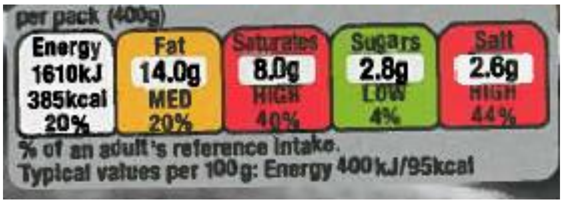

Meal 2 (Chicken & Veg Sizzler)

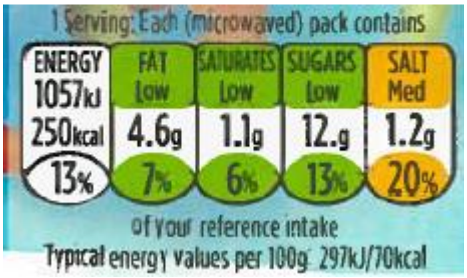

Both meals are under 400kcal, but meal 1 is high in saturated fat and salt (2 reds), whereas meal 2 is low in saturated fat and medium in salt content – more greens. You might choose meal 1 occasionally and meal 2 more often.

5. Work out the number of portions in the meal

This can be done by eye (e.g. estimating the number of slices of bread and amount of filling in a sandwich), or can be worked out more accurately from the ingredients list on the pack. To do this:

i. Find the ingredients list on the pack. Ingredients are listed in descending order, so the first ingredient makes up most of the weight of the product and the last ingredient on the list makes up the smallest amount.

ii. The list will give you the proportions of the key ingredients.

For example:

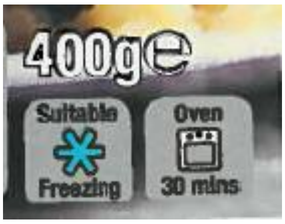

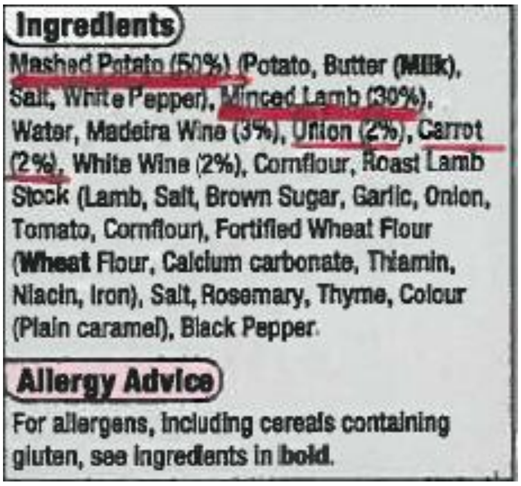

Ingredients

Mashed Potato (50%) (Potato, Butter (**Milk**), Salt, White Pepper), Minced Lamb (30%), Water, Madeira Wine (3%), Onion (2%), Carrot (2%), White Wine (2%), Cornflour, Roast Lamb Stock (Lamb, Salt, Brown Sugar, Garlic, Onion, Tomato, Cornflour), Fortified Wheat Flour (**Wheat** Flour, Calcium carbonate, Thiamin, Niacin, Iron), Salt, Rosemary, Thyme, Colour (Plain caramel), Black Pepper

Allergy Advice

For allergens, including cereals containing gluten, see ingredients in **bold**.

Ignore ingredients in brackets, as these just tell you what is in each of the main ingredients. For example, Mashed Potato (50%) (Potato, Butter (**Milk),** Salt, White Pepper) tells you that the mash is made up of the ingredients in brackets.

We are only interested in the main ingredients.

iii. Work out the weight of each of the main ingredients (ignore herbs, spices, preservatives etc.). This product weighs 400g. From the label:

Mash (starchy food) = 50% of the whole product which is 200g

Lamb (protein food) is 30% of the whole product which is about 65g

Carrot and onion (veg) make up 4% of the product. Working out 10% (40g) and halving this (20g) will give about 5% (accurate enough!) So 20g is veg.

iii. Compare these to the portion sizes on pages 5-8:

1 portion potato = 150g, so there are about 1.5 portions in the meal (200g).
1 portion lamb = 60g, so there is 1 portion in the meal.
1 portion veg = 80g, so there is only ¼ portion in the meal.

iv. Use my fitness pal for any foods not in the tables. You can work out the number of portions from the kcal (1 starchy = 100kcal etc).

Managing Hunger

You may worry that you will feel hungry while following the diet plan. The following tips will help you reduce and manage hunger:

1. **If you feel hungry, try drinking a pint of water**
2. Make sure you are eating all of your food portion allowances each day.
3. **A bowl of soup or salad before a meal helps to fill you up and is low in calories.**
4. Choose fewer refined carbohydrates (made from processed white flour and sugars) and more of the starchy foods which keep you fuller for longer – see the box on the bottom of page 3 for suggestions.
5. **Use a smaller bowl and plate. Your plate will look fuller and you will feel more satisfied after a meal.**
6. Eat slowly to give ‘fullness’ signals a chance to reach your brain. If you eat quickly or on the go, you won’t start to feel full until 20 minutes after you have finished.
7. **Focus on what you are eating. If you are doing other things like watching TV, working or reading you won’t appreciate what you have eaten and won’t feel so satisfied.**
8. Hunger is often confused with cravings. If you have eaten a meal in the last 2 hours, you are unlikely to be experiencing true hunger. Try distraction techniques like going for a walk, reading or having a bath – anything that will occupy you and take your mind off food. Acknowledge

that you *could* eat but you aren't going to this time. Cravings will eventually die away, so persevere and delay responding to them.

9. **If you are still hungry, try the snack ideas on page 9**
10. Remember, hunger is a positive sign that the diet plan is working!

Plan a day's intake

**FOOD & DRINKS**	**STARCHY FOODS**	**PROTEIN FOODS**	**DAIRY**	**FRUIT**	**VEGETABLES**	**FATS/OILS**
Breakfast						
Mid-Morning						
Lunch						
Mid Afternoon						
Evening Meal						
Evening Snacks/Supper						
**(Portions in Plan)**<br>**TOTAL PORTIONS:**	**(4)**	**(2)**	**(2)**	**(1)**	**(4)**	**(2)**

WELLBABE Study Contacts:

Dr Ken Hodson

Email:

Phone:

Alison Barnes (study dietitian)

Email:

Phone:

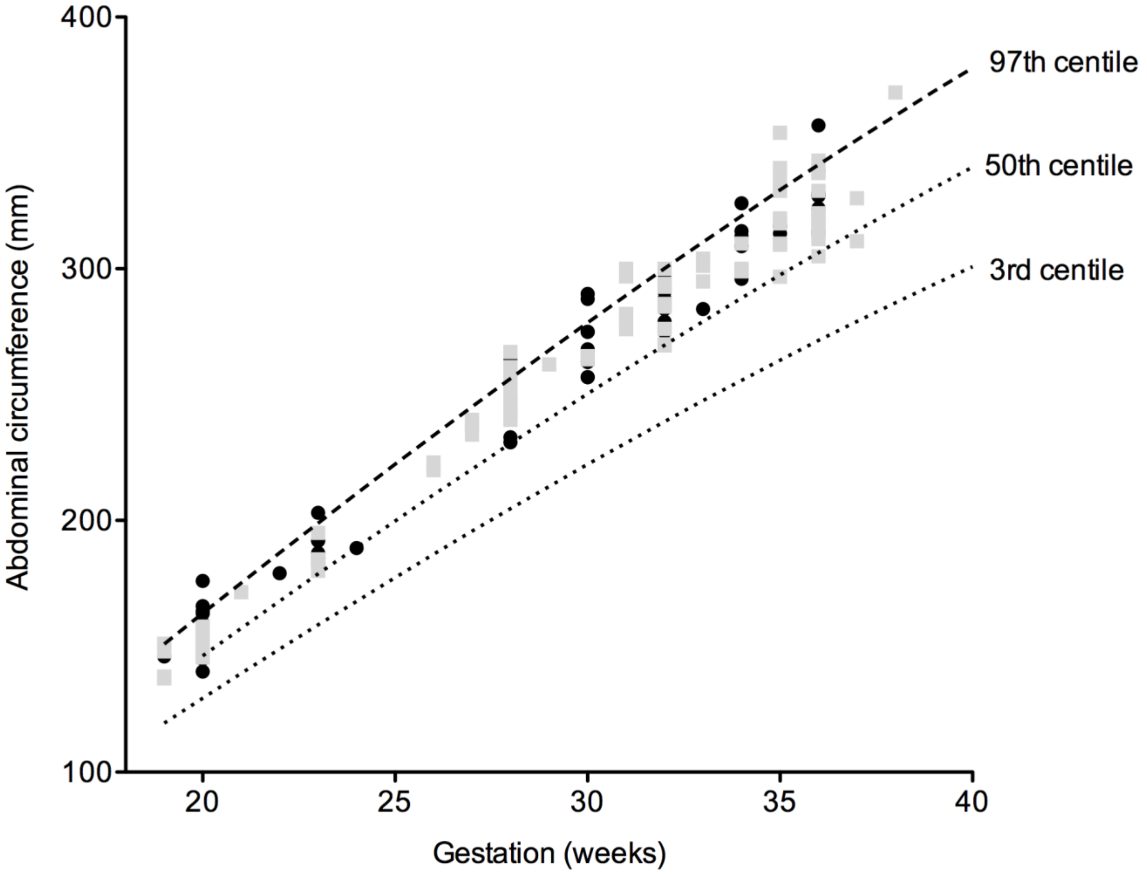

ESM Figure 1 Fetal abdominal circumference of study participants (black circles) and controls (grey circles).
